# A Superior Corrosion Protection of Mg Alloy via Smart Nontoxic Hybrid Inhibitor-Containing Coatings

**DOI:** 10.3390/molecules28062538

**Published:** 2023-03-10

**Authors:** Andrey S. Gnedenkov, Valeriia S. Filonina, Sergey L. Sinebryukhov, Sergey V. Gnedenkov

**Affiliations:** Institute of Chemistry, Far Eastern Branch of the Russian Academy of Sciences, 159 Pr. 100-letiya Vladivostoka, Vladivostok 690022, Russia

**Keywords:** magnesium alloy, anticorrosion protection, plasma electrolytic oxidation (PEO), corrosion inhibitor, 8-hydroxyquinoline, polycaprolactone, corrosion mechanism

## Abstract

The increase of corrosion resistance of magnesium and its alloys by forming the smart self-healing hybrid coatings was achieved in this work in two steps. In the first step, using the plasma electrolytic oxidation (PEO) treatment, a ceramic-like bioactive coating was synthesized on the surface of biodegradable MA8 magnesium alloy. During the second step, the formed porous PEO layer was impregnated with a corrosion inhibitor 8-hydroxyquinoline (8-HQ) and bioresorbable polymer polycaprolactone (PCL) in different variations to enhance the protective properties of the coating. The composition, anticorrosion, and antifriction properties of the formed coatings were studied. 8-HQ allows controlling the rate of material degradation due to the self-healing effect of the smart coating. PCL treatment of the inhibitor-containing layer significantly improves the corrosion and wear resistance and retains an inhibitor in the pores of the PEO layer. It was revealed that the corrosion inhibitor incorporation method (including the number of steps, impregnation, and the type of solvent) significantly matters to the self-healing mechanism. The hybrid coatings obtained by a 1-step treatment in a dichloromethane solution containing 6 wt.% polycaprolactone and 15 g/L of 8-HQ are characterized by the best corrosion resistance. This coating demonstrates the lowest value of corrosion current density (3.02 × 10^−7^ A cm^−2^). The formation of the hybrid coating results in the corrosion rate decrease by 18 times (0.007 mm year^−1^) as compared to the blank PEO layer (0.128 mm year^−1^). An inhibitor efficiency was established to be 83.9%. The mechanism of corrosion protection of Mg alloy via smart hybrid coating was revealed.

## 1. Introduction

The development of biomedical technologies is aimed, in particular, at accelerating the reparative osteogenesis of an injured bone and minimizing the health damage during the healing process. The problem of using traditional non-resorbable implants is caused not only by the need for reoperation to remove them but, as a rule, is associated with allergic reactions, the release of toxic ions or wear microparticles of the implanted material, as well as the insufficient biological and physical-chemical bond between the implant and bone tissue [1,2]. Considering the duration of a foreign object’s presence in the human body, the implant material components should not harm it. Among the variety of metallic biomaterials, magnesium and its alloys are not only among the most studied at present but also attract the greatest interest from a practical point of view [3]. According to [1,2,3,4], the main advantages of magnesium over other metal implant materials are high specific strength with low weight, high damping capacity, biocompatibility, and also its bioresorption. Since Young’s modulus for magnesium and its alloys is close to the value of this parameter for human bone [2,5], the use of magnesium implants makes it possible to minimize the mechanical stresses at the bone-implant interface [6,7,8,9]. The main problem with the use of magnesium and its alloys is their intense corrosion, accompanied by the release of gaseous hydrogen during an interaction with environments [3,9,10,11]. The best properties will be possessed by implants characterized by both a degradation rate corresponding to the rate of bone healing and bioactive properties accelerating osteogenesis. The most common ways to improve the electrochemical properties of bioresorbable magnesium alloys are mechanical treatment by plastic deformation (extrusion, pressing, twisting, etc.) [5,12,13,14,15,16], alloying [16,17,18,19,20,21,22,23], and formation of protective bioactive coatings. Several methods are known for applying such protective layers, for example, sol-gel technology [24,25], layer-by-layer deposition (polyelectrolyte multilayers) [26,27], hydrothermal deposition [28,29], plasma spraying [30,31], laser cladding [32], electrochemical [33,34,35,36] and chemical [37,38,39] deposition, cold sprayed deposition, etc. [40]. However, these methods have disadvantages, such as process duration, the need for multiple applications, in some cases, the shrinkage of the final coating, as well as the use of a large number of solvents.

One of the optimal ways to form the coating on a magnesium alloy surface is plasma electrolytic oxidation (PEO) [41,42,43,44,45,46]. As a result of modification by this method, a strong ceramic-like coating is formed on the alloy surface. The advantage of the PEO method is its manufacturability, environmental friendliness, and versatility. Unlike other methods of applying bioactive coatings, the growth of the protective PEO layer occurs in two directions at the electrode/electrolyte interface, which ensures increased adhesion of the formed coating to the metal substrate [47,48,49]. It is known that calcium phosphate PEO coatings have the best biocompatibility due to their composition being similar to the inorganic components of human bone [41]. The composition and properties of formed coatings directly depend on the electrolyte composition and oxidation mode [50,51].

However, taking into account the period of complete healing of bone tissue (14–17 weeks), the level of protective properties of calcium phosphate PEO coatings is not enough to provide the necessary corrosion resistance during the entire rehabilitation period. The penetration of human body fluids to the metal substrate through the pores and microdefects of the coating structure (areas of initiation of corrosion processes) can lead to a significant increase in metal electrochemical activity and further destruction of the implant.

At the same time, the described coating morphology makes it possible to modify the oxide layer with various protective agents that contribute to a significant improvement in the corrosion properties of the product. An improvement in the corrosion resistance of a PEO coating can be reached through the impregnation of its porous part with corrosion inhibitors [52,53]. Chemical substances capable of reducing the electrochemical activity of a material include chelating agents [53,54], one of which is 8-hydroxyquinoline (8-HQ), used in the formation of self-healing coatings, which is considered the most popular.

It was shown in [55] that the formation of crystalline structures on the surface of a PEO layer after inhibition by 8-hydroxyquinoline promotes an increase in surface homogeneity, which indicates the distribution of the chelating agent in pores and microdefects. Taking into account the high porosity of the base PEO layer, it can be concluded that such “reservoirs” have sufficient capacity for inhibitor loading. It was shown in [54,56] that the chemical interaction of the inhibitor with magnesium ions begins after PEO coating damage as a result of the corrosion process. Pitting areas acting as local anode and cathode zones are characterized by a change in local pH values, which enhances the inhibitor solubility and makes it possible to interact with magnesium ions (self-healing effect). Taking into account these features and according to [52,57], the porous inhibitor-loaded PEO coatings can provide a self-healing effect.

An analysis of the results obtained by various scientific groups indicates the possibility of forming inhibitor-containing coatings based on multifunctional layers. For example, the work [58] shows that impregnation of layered double hydroxide coatings on AZ31 magnesium alloy with 8-hydroxyquinoline promotes higher corrosion resistance even after 30 days of exposure to a 3.5% NaCl solution, in comparison with a coating without inhibitor. A comparative analysis of the properties of PEO layers formed on an AZ31 magnesium alloy in an alkaline silicate electrolyte without and with the addition of ZrO_2_ nanoparticles was carried out [55]. It was established a significant improvement in the corrosion resistance of the nanoparticles-free PEO-coated samples after immersion in 0.05 M solution of 8-HQ for 72 h due to the formation of flower-like structures of magnesium 8-hydroxyquinalinate (Mg(8-HQ)_2_). The work [59] dealt with a study of surface layers obtained on a WE42 magnesium alloy by a combination of PEO and 8-HQ treatment. It was established that an increase in the 8-HQ concentration and exposure time correlates with a significant increase in the electrochemical stability of the resulting surface layers, which is caused by an increase in the level of protective properties due to the interaction of the organic and inorganic parts of the coating. The authors also suggested that the Mg(8-HQ)_2_ crystalline structures formed on an inorganic surface can be used in biosensors, antioxidants, bioanalytical devices, and industrial catalysis. The aim of the study [60] was to form on AZ31 Mg alloy the PEO layers with the addition of TiO_2_ and SnO_2_ nanoparticles in the electrolyte and subsequent treatment in inhibitor-containing solutions. As a result of the research, it was revealed that the 8-hydroxyquinoline loading enhances the photocatalytic and reduces the electrochemical activity of the obtained coatings.

8-hydroxyquinoline is the most common quinoline compound in medicinal chemistry, which is a matrix with a wide range of pharmacological actions [61,62,63,64,65,66,67,68,69,70] and has many other properties. 8-HQ refers to the so-called privileged structures, which are currently being actively investigated as promising active substances or potential drugs [71,72,73]. The broad range of pharmacological applications of 8-HQ derivatives is presented in [71,74,75]. 8-HQ-based compounds can serve as neuroprotection agents (HLA20, VK28, M30, Clioquinol, PBT2, DPH6, LA-HQ-LA, GS(HQ)H, D-369, D-390), anticancer agents (NSC3852, JLK 1472, JLK 1486, S1, nitroxoline), anti-HIV agents (styrlquinoline FZ149), etc. [71]. Based on the presented data, it can be concluded that 8-HQ is not only harmless to the human organism but can also act as a potent drug candidate for the treatment of various diseases.

One of the currently widespread methods for reducing the degradation rate of bioresorbable magnesium alloys is a surface modification with biodegradable polymeric materials [76]. Such coatings differ from their inorganic counterparts in many aspects in providing protective functions. Biodegradable polymers are used in biomedicine, for example, as components of antibacterial coatings, surgical sutures, drug delivery systems, fixation devices, and tissue replacement components [77,78]. Polymeric materials are considered promising tools for controlling cell adhesion, proliferation, and differentiation [79]. Thus, further study of the properties and methods of applying biodegradable polymeric materials, as well as the possibility of their combination with other protective technologies, will significantly expand the use of magnesium and its alloys in biomedicine. Among other synthetic biopolymers, polycaprolactone (PCL) has excellent physicochemical properties [80], including biodegradability [81], biocompatibility [82], structural stability [83], low melting point, and elasticity [84].

The current scientific literature data describe various methods for applying polycaprolactone, particularly to biomedical products. The authors of [85] studied the properties of coatings obtained by plasma electrolytic oxidation of commercially pure magnesium in a silicate-fluoride electrolyte followed by treatment with PCL. As a result of immersing the samples in the Hanks solution, a positive effect of the polymer material component on the corrosion resistance of the surface layer was revealed. The authors of [86] obtained a two-layer coating on an AZ31 Mg alloy treated with hydrofluoric acid, consisting of a PCL matrix modified with hydroxyapatite nanoparticles and deposited on a thin layer of polyesterimide. It was revealed that the presence of hydroxyapatite in the coating composition affects both its corrosion resistance and cell behavior, promoting the differentiation of osteoblasts.

A method for the formation of nanosized PCL filaments by electrospinning was developed in [87]. The authors of [88] presented the ultrasonic spray method for obtaining a coating containing PCL and polylactide, which was optimized for bioresorbable vascular stents made of AZ31 magnesium alloy. In [89], the authors concluded that surface modification of the AZ91 Mg alloy using PCL-containing coatings obtained using the sputtering method helps to reduce the corrosion damage of the bioresorbable material. In [90], a positive effect of PCL film formation by spin coating on corrosion properties and cytocompatibility of commercially pure Mg was reported. The authors of [91] showed that PCL structures obtained on AM50 Mg alloy samples by the dip-coating technique contribute to high adhesion and proliferation of osteoblasts.

Based on the analysis of the relevant literature data, it can be concluded that the organic inhibitor 8-hydroxyquinoline and biodegradable polymer polycaprolactone have a positive effect on the corrosion resistance of various types of coatings, in particular, formed by plasma electrolytic oxidation. However, the complex action of these protective agents has not been previously established and studied.

In this present work, a new method is proposed for controlling the corrosion degradation of bioresorbable magnesium alloys by forming hybrid bioactive coatings based on a calcium- and phosphate-containing oxide layer with a self-healing function.

## 2. Results and Discussion

### 2.1. Coatings’ Structure and Composition

**Figure 1:**
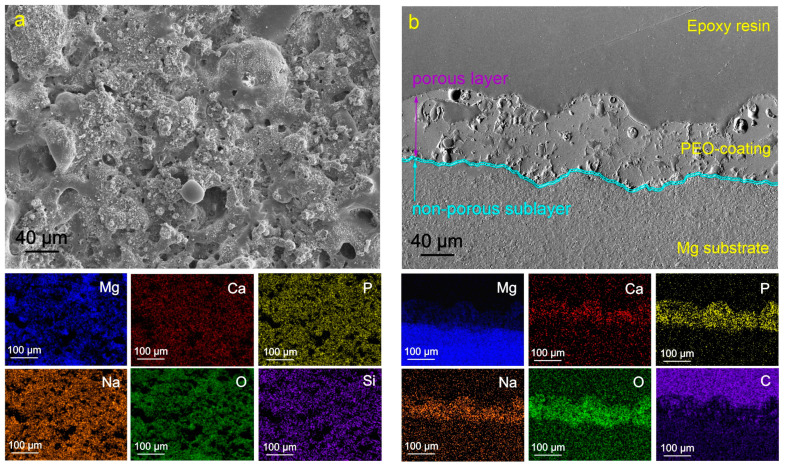
SEM images of the surface (**a**) and cross-section (**b**) of the PEO-coated samples and maps of elements distribution over the sample’s surface (**a**) and thickness (**b**).

As a result of plasma electrolytic oxidation, a ceramic-like coating was formed on the magnesium alloy surface (Figure 1). Based on the data of X-ray phase analysis, it was previously revealed [52] that the composition of obtained surface layers is characterized by the presence of magnesium oxide (MgO), magnesium orthosilicate Mg_2_SiO_3_, magnesium-sodium metasilicate Na_2_MgSiO_3_, as well as calcium phosphate (Ca-P) compounds, including hydroxyapatite (Ca_10_(PO4)_6_(OH)_2_). The heterogeneity of surface morphology, expressed by the presence of pores and microdefects, contributes to further impregnation of the coating with inhibitor and subsequent processing with a polymeric material.

**Figure 2:**
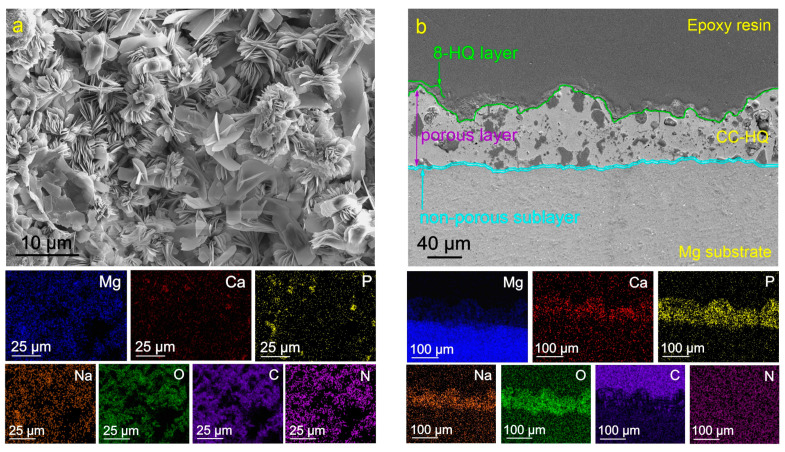
SEM images of the surface (**a**) and cross-section (**b**) of the CC-HQ-coated samples and maps of elements distribution over the sample’s surface (**a**) and thickness (**b**).

The impregnation of the oxide layer with 8-HQ leads to the formation of crystalline structures on the sample surface (Figure 2a). Based on the data on the distribution of elements over the surface and thickness of the sample (Figure 2), the composition of the formed layer is characterized by the presence of nitrogen (N), oxygen (O), and carbon (C). These elements are included in the composition of the 8-HQ (C_9_H_7_NO) compound. The distribution of these elements corresponds to the zones with a higher concentration of magnesium and sodium (Figure 2). An analysis of the results indicates a possible formation of magnesium 8-hydroxyquinalinate (Mg(8-HQ)_2_) and sodium 8-hydroxyquinalinate (Na(8-HQ)) compounds as a result of coating treatment with a corrosion inhibitor. The obtained data do not contradict the results of [55,58,59].

**Figure 3:**
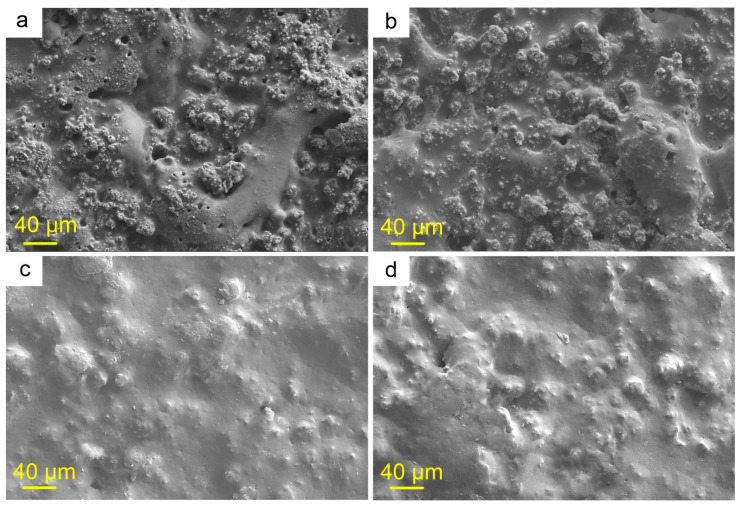
SEM images of PEO-coated samples treated with a 3 wt.% (**a**,**b**) and 6 wt.% (**c**,**d**) solution of polycaprolactone in acetone (**a**,**c**) and dichloromethane (**b**,**d**).

The next stage of this study was to determine the optimal concentration of polycaprolactone in solutions and to select the simplest and most effective method for impregnating the porous PEO layer. Double processing with a 3 wt.% solution of PCL in 2 studied solvents contributed to an increase in the homogeneity of the coating (Figure 3a,b) compared to the base PEO layer (Figure 1). However, this increase in homogeneity is not enough to ensure maximum corrosion resistance due to the presence of unfilled pores with polymeric material. Subsequently, these areas can probably become centers of initiation and development of corrosion processes, contributing to the premature start of the self-healing process, and, consequently, to a faster end of the inhibitory effect [52]. As a result, the long-term provision of protective properties will probably not be achieved. The treatment of a PEO coating with 6 wt.% solutions of polycaprolactone in dichloromethane (CC-D) and acetone (CC-A) contributed to a significant increase in the homogeneity of the surface relief and a significant decrease in the number of pores and microdefects (Figure 3c,d). This concentration of polymeric material in solutions was accepted as the most suitable for further studies.

**Figure 4:**
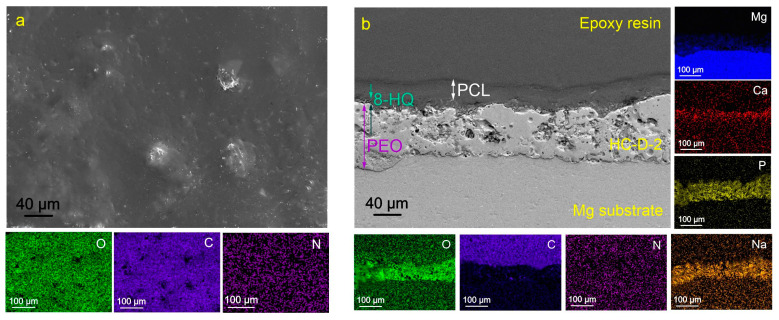
SEM images of the surface (**a**) and cross-section (**b**) of the HC-D-2-coated samples and maps of elements distribution over the sample’s surface (**a**) and thickness (**b**).

**Figure 5:**
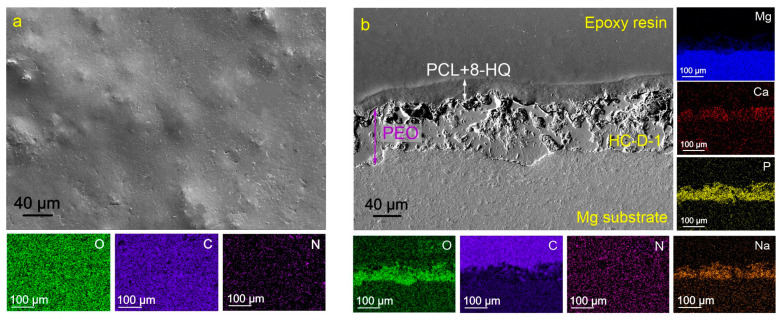
SEM images of the surface (**a**) and cross-section (**b**) of the HC-D-1-coated samples and maps of elements distribution over the sample’s surface (**a**) and thickness (**b**).

**Figure 6:**
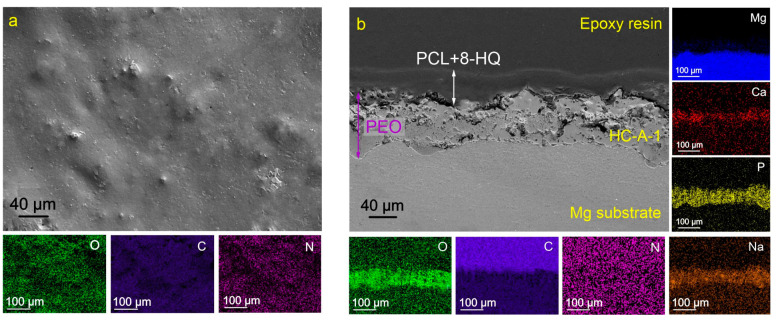
SEM images of the surface (**a**) and cross-section (**b**) of the HC-A-1-coated samples and maps of elements distribution over the sample’s surface (**a**) and thickness (**b**).

Figure 4, Figure 5 and Figure 6 show the morphological structures and maps of the element distribution over the surface of HC-D-2, HC-D-1, and HC-A-1 samples. The surface of the presented coatings is more homogeneous in comparison with the base PEO layer and the inhibitor-containing coating CC-HQ. Hybrid surface layers contain a high concentration of oxygen (O) and carbon (C), which are the main components of polycaprolactone [–(CH_2_)_5_–CO–O–]_n_. From the results of the analysis of cross-sections of the obtained coatings, it can be concluded that the impregnation of the PEO coating matrix with polymeric material contributes to the complete filling of the pores of the oxide layer. It should be noted that the proposed method of polycaprolactone application makes it possible to obtain a uniform surface layer.

**Figure 7:**
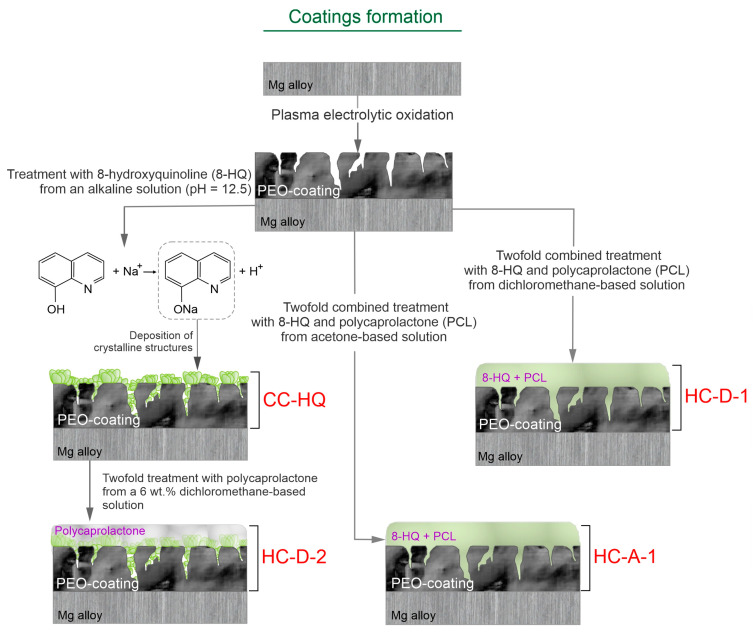
The detailed process of the base and inhibitor-containing coatings’ formation.

For a better interpretation of the results of this study, the detailed process of the base and inhibitor-containing coatings’ formation (PEO-coated, CC-HQ, HC-D-1, HC-D-2, and HC-A1 samples) and the content of the obtained protective layers are summarized in Figure 7.

### 2.2. Electrochemical Properties

**Figure 8:**
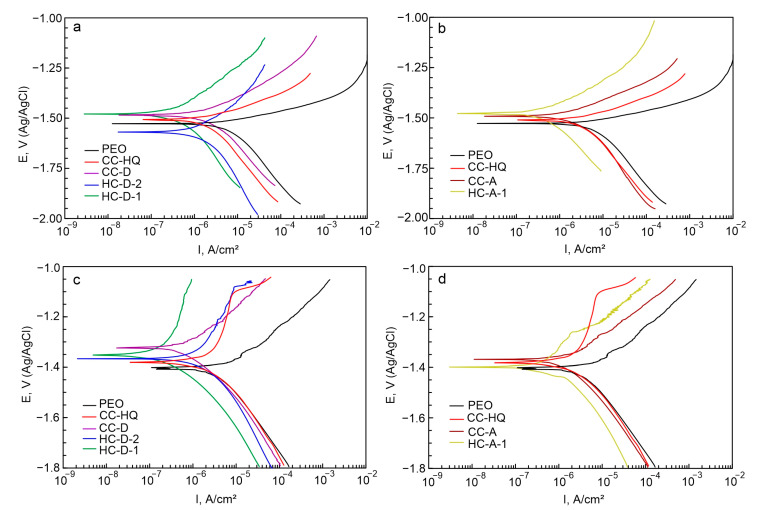
Polarization curves obtained after 10 min (**a**,**b**) and 22 h (**c**,**d**) exposure of samples with various coatings in 3.5% NaCl solution.

The results of the preliminary estimation of electrochemical properties of the formed surface layers (after 10 min sample exposure to 3.5 wt.% NaCl aqueous solution) by the PDP method are shown in Figure 8a,b. Based on the results of a comparative analysis of the calculated parameters, it can be assumed that hybrid coatings exhibit the best corrosion resistance among the studied surface layers. It was revealed that the corrosion current density, *I_c_*, for HC-D-2 (4.99 × 10^−7^ A/cm^2^) and HC-D-1 (3.02 × 10^−7^ A/cm^2^) are lower by 3 and 5 times, respectively, as compared to CC-D (1.52 × 10^−6^ A/cm^2^). HC-A-1 sample is characterized by more than a 3-fold decrease in the values of this parameter in comparison with CC-A (5.20 × 10^−7^ A/cm^2^ and 1.74 × 10^−6^ A/cm^2^, respectively) (Table 1). The electrochemical behavior of samples after long-term exposure to an aggressive corrosive environment (after 22 h sample exposure to 3.5 wt.% NaCl aqueous solution) was also studied by the PDP method (Figure 8c,d). The data presented in Table 1 and Figure 8c,d allow us to conclude that after a long interaction of the material with a corrosive environment, hybrid coatings also demonstrate the best protective characteristics. Thus, the decrease in *I_c_* values for HC-D-2 (3.52 × 10^−7^ A/cm^2^) and HC-D-1 (1.64 × 10^−7^ A/cm^2^) was 3 and 6 times, respectively, compared to the values for CC-D (1.02 × 10^−6^ A/cm^2^). The values of corrosion current density for HC-A-1 (6.37 × 10^−7^ A/cm^2^) were more than 2 times lower than for CC-A (1.77 × 10^−7^ A/cm^2^). Analysis of the values of the polarization resistance (*R*_P_) confirmed the increase of the protective properties after hybrid coating formation (Table 1). HC-D-1 sample has the highest value of the *R*_P_ (1.06 × 10^5^ Ω·cm^2^), which is 35 times higher than for the base PEO layer (3.05 × 10^3^ Ω·cm^2^).

It should be noted that values of the corrosion potential (*E_c_*) increased during the 22 h of immersion in the corrosive medium (from ca. −1.5 up to −1.3 in Table 1 and Figure 8). This result is related to the change in the morphology and composition of the initial coatings as a result of their partial degradation and formation of the corrosion product film as well as to the self-healing effect of the inhibitor-containing layers [42,52].

For the samples with composite polymer-containing coatings (CC-D, CC-A), an intensive increase in current density in the potential range between −1.3 V and −1.0 V was established. This is the result of the degradation of the formed protective layers and the penetration of an aggressive medium to the material substrate (Figure 8c). The dynamics of change in the current density of the anodic curve for HC-D-1 is characterized by a sharp decrease in growth intensity of this value after 22 h of immersion in a corrosive environment. This indicates the manifestation of self-healing properties (Figure 8c). It should be noted that this tendency was not observed for HC-D-2 and HC-A-1 samples. In this case, as a result of the long-term interaction of the coating with NaCl solution, pitting occurs on the samples’ surface. This is associated with a lower concentration of the inhibitor loaded during the formation of the hybrid layer HC-D-2 (8-HQ concentration was 3 g/L) as compared to HC-D-1 (8-HQ concentration was 15 g/L), as well as with partial dissolution of 8-hydroxyquinoline in dichloromethane during the deposition of the polymer component. The higher corrosion activity of the HC-A-1 sample (compared to HC-D-1) is due to the lower solubility of polycaprolactone in acetone (in comparison with dichloromethane). This leads to a lower concentration of the polymer in the coating composition, as well as to lower compactness and lower protective properties of the formed surface film formed in acetone (in comparison with one formed in dichloromethane).

The highest values of the corrosion rate (*CR*), calculated on the basis of the PDP data, were detected for the PEO coating (0.128 mm year^−1^). Modification of the PEO layer with a polymer and inhibitor promotes the decrease in the *CR*. The lowest *CR* was established for hybrid coatings. The maximum decrease (18 times as compared to the blank PEO layer) in the corrosion rate was detected for the HC-D-1 sample (0.007 mm year^−1^).

**Figure 9:**
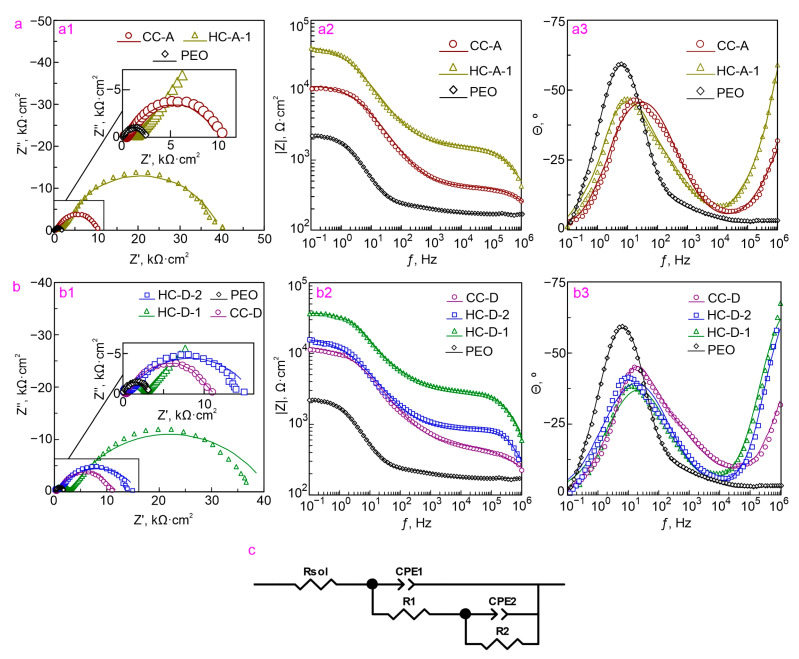
Impedance spectra Nyquist (**a1**,**b1**) and Bode plots (**a2**,**a3**,**b2**,**b3**) for the studied samples acquired after 10 min of exposure to 3.5 wt.% NaCl solution (**a**,**b**) and equivalent electrical circuit (**c**) used to fit the experimental data.

**Figure 10:**
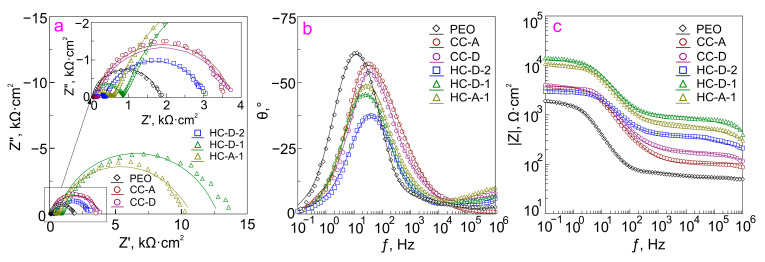
Impedance spectra Nyquist (**a**) and Bode plots (**b**,**c**) for the studied samples acquired after 22 h of exposure to 3.5 wt.% NaCl solution.

Determining the level of protective properties of samples with composite and hybrid coatings at the initial stage and after the long-term process of immersion (10 min and 22 h, respectively) are shown by comparing the impedance spectra presented in the form of Nyquist and Bode plots (Figure 9a,b and Figure 10). Dependencies of phase angle *θ* on frequency *ƒ* for PEO, HC-D-2, HC-D-1, HC-A-1, CC-D, and CC-A samples are characterized by the presence of two-time constants (Figure 9 and Figure 10). In this regard, the fitting of the impedance spectra was carried out using an equivalent electrical circuit (EEC) with two series-parallel connected *R-CPE* chains (Figure 9c). In this work, a constant phase element (*CPE*) was used instead of the ideal capacitance due to the high heterogeneity of studied surface layers. Elements *R*_1_ and *CPE_1_* characterize the resistance of the porous part of the PEO coating (outer layer) impregnated with a polymer (CC-D and CC-A) or polymer and inhibitor (HC-D-2, HC-D-1, and HC-A-1), and the “geometric” capacitance of the whole coating. *R*_2_*–CPE*_2_ chain represents the resistive and capacitive components of the non-porous sublayer of the PEO coating (inner layer), including compounds formed as a result of the interaction of the inhibitor and the polymer with the material of the inner part of the PEO layer.

The evolution of the calculated parameters of EEC elements for samples with different types of coatings formed on the MA8 magnesium alloy during 22 h of exposure to 3.5% NaCl solution is presented in Table 2 and Table S1–S3. Parameters *Q* and *n* (*CPE* constant and exponential factor, respectively) during the immersion of the sample vary slightly, which indicates a slight change in the morphology, composition, and properties of the coatings. Such possible processes as an increase/decrease in the thickness of the protective layer, degradation of the coating, formation of a protective film during self-healing, and formation of corrosion products in the defect zone can contribute to the change in *Q*. A significant increase in the resistance of the outer (*R_1_*) and inner sublayers of the coating (*R*_2_) is due to impregnation of the coating with a corrosion inhibitor and polymer. During the immersion of the sample in a corrosive environment, the parameters *R*_1_ and *R*_2_ decrease due to partial degradation of the protective layer. Nevertheless, HC-D-1 and HC-A-1 samples demonstrate high protective characteristics even after 22 h of immersion in a chloride-containing environment (Table 2, Table S2, and Table S3).

A significant increase in the diameter of the semicircle on the complex plane for HC-D-1 and HC-A-1 samples after 10 min of exposure, as compared to the corresponding diameter for samples with CC-D and CC-A, also indicates a clear advantage of hybrid coatings (Figure 9). The value of the impedance modulus measured at a low frequency (*|Z|*_*f*=0.1__Hz_) for the HC-D-1 sample (105,350 Ω·cm^2^) is by 1 order of magnitude higher than the value of this parameter for the CC-D sample (10,578 Ω·cm^2^) and for the HC-A-1 sample. The value *|Z|*_*f*=0.1__Hz_ (54,074 Ω·cm^2^) for the HC-A-1 sample is characterized by a more than 4-fold increase in the corresponding value obtained for CC-A (11,318 Ω·cm^2^) (Table 1).

A higher diameter of the semicircle on the complex plane, as well as a higher impedance modulus measured on the whole frequency range, remained for HC-D-1 and HC-A-1 specimens after 22 h of exposure, as compared to other types of protective layers (Figure 10). *|Z|*_*f*=0.1__Hz_ for HC-D-1 and HC-A-1 samples (12,995 Ω·cm^2^ and 10,167 Ω·cm^2^, respectively) is still higher than the value of this parameter for CC-D (3817 Ω·cm^2^) and CC-A (3712 Ω·cm^2^) specimens (Table 1).

An analysis of the obtained data enables one to conclude that the incorporation of an inhibitor into the composite polymer-containing coatings based on a calcium phosphate layer formed by the PEO method contributes to a significant decrease in the electrochemical activity of magnesium alloy samples due to the function of active corrosion protection. Based on the analysis of PDP data (Figure 8, Table 1), an HC-D-1 sample demonstrates the highest anticorrosive protection among the presented samples. This type of coating is characterized by the lowest value of corrosion current density (after 10 min and 22 h exposure to 3.5% NaCl *I_c_* = 3.02 × 10^−7^ A/cm^2^ and 1.64 × 10^−7^ A/cm^2^, respectively).

**Figure 11:**
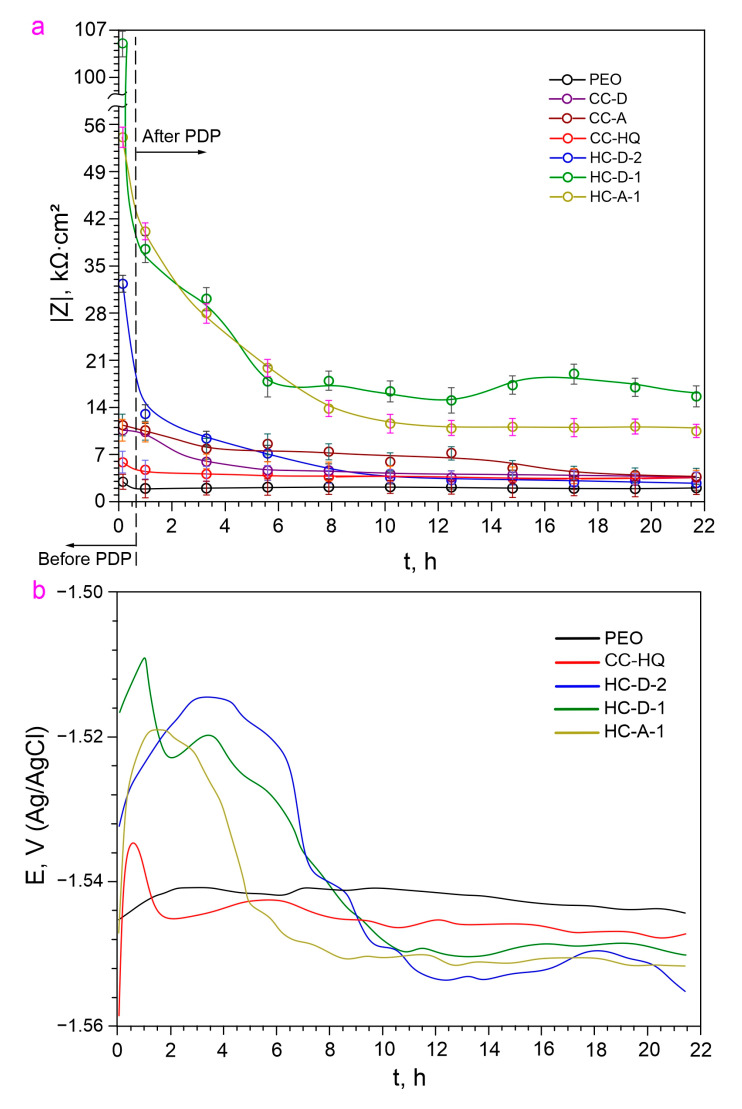
Evolution of impedance modulus measured at a low frequency (**a**) and open circuit potential (**b**) for studied samples during 22 h of exposure to 3.5 wt.% NaCl solution.

Based on the data on the dynamics of change in the impedance modulus measured at a low frequency (*|Z|*_*f*=0.1__Hz_) for the studied samples (Figure 11a), it can be concluded that the HC-D-1 sample possesses the best corrosion resistance in the studied medium during the whole exposure time. This is confirmed by the highest value of the impedance modulus after 5 h of exposure to 3.5% NaCl solution, as well as the dynamics of its change, indicating the manifestation of self-healing ability (in the period of 8–18 h of exposure, alternating increasing and decreasing stages of *|Z|*_*f*=0.1__Hz_ are observed). The self-healing effect is based on the formation of the protective layer on the surface of the defect zone after the coating breakdown. This protective film formed as a result of corrosion inhibitor action prevents the intensive degradation of the material. However, since this newly formed layer is not the initial PEO coating and has a lower protective ability, the EIS results cannot show the increase of the anticorrosion characteristics up to the ones measured before the coating breakdown. This protective layer formed on the defect in inhibitor-containing coating provides the Mg alloy with better protection against corrosion as compared with inhibitor-free coating systems. Therefore, one can see the lower deterioration rate for the inhibitor-containing layers especially for the hybrid coatings as compared to the blank PEO layers (Figure 11a).

One can find some deviations in the parameters measured using PDP and EIS, which resulted in a low correlation of the corrosion current density and calculated corrosion rate with the impedance modulus evolution (Table 1). This can be the result of different methodic of these electrochemical techniques. The polarization effect, which is realized during PDP measurements, can intensify the sample degradation rate and ensure a higher rate of corrosion product formation compared to this process during the EIS test which operated at OCP. Moreover, polarization can change the corrosion inhibitor activity and increase the self-healing effect of the samples with 8-HQ-containing coatings. Therefore, the results obtained using PDP measurements show a higher protective ability of the samples after 22 h immersion compared to the corrosion resistance evaluated from EIS data. Corrosion current density and values of the corrosion rate are slightly changed compared to the impedance modulus measured at the lowest frequency. The discrepancy in the EIS and PDP data was shown in the previous work [80]. However, the HC-D-1 sample has the best protective properties according to EIS and PDP measurements (Table 1 and Table 2).

The analysis of the evolution of the open circuit potential during the exposure time of the sample in a 3.5 wt.% NaCl solution (Figure 11b) indicates a general trend towards a decrease in the value of this parameter after 8 h of exposure. This is the result of the gradual penetration of the chloride-containing medium into the material substrate. The nature of the open circuit potential decrease for HC-D-1 indicates longer protection of the material against corrosion damage in comparison with other samples.

**Figure 12:**
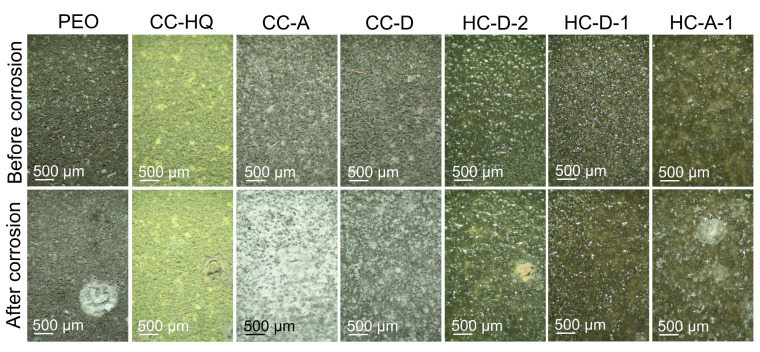
The photographs of the samples before and after 7 days of immersion in 3.5 wt.% NaCl solution.

In order to prove the self-healing effect of the inhibitor-containing coating, the 7-day exposure test was performed. Analysis of the photographs of the samples before and after immersion in 3.5 wt.% NaCl solution presented in Figure 12 clearly shows the better appearance of the HC-D-1 specimen. This sample with hybrid coating has no evident pitting formed during exposure to an aggressive medium. The HC-A-1 specimen has a lower area of corrosion degradation compared to specimens with the blank PEO coating, composite layers, and HC-D-2. The severe corrosion degradation was registered only for the inhibitor-free PEO coating. Other types of coated samples show higher coating integrity. Small pitting areas were also revealed on the surface of the CC-D sample.

### 2.3. Inhibitor Efficiency Estimation

The calculation of inhibitor efficiency (*η_i_*) of the formed hybrid coatings (Table 3) allowed us to reveal that HC-D-1 samples are characterized by the highest level of corrosion protection. *η_i_* value for this type of coating at the initial stage of corrosion studies was 80.1%. After sample polarization and long-time EIS studies in 3.5 wt.% NaCl solution, an inhibitor efficiency increased up to 83.9%, which indicates the most pronounced manifestation of self-healing properties (Table 3).

### 2.4. Wear Resistance

**Figure 13:**
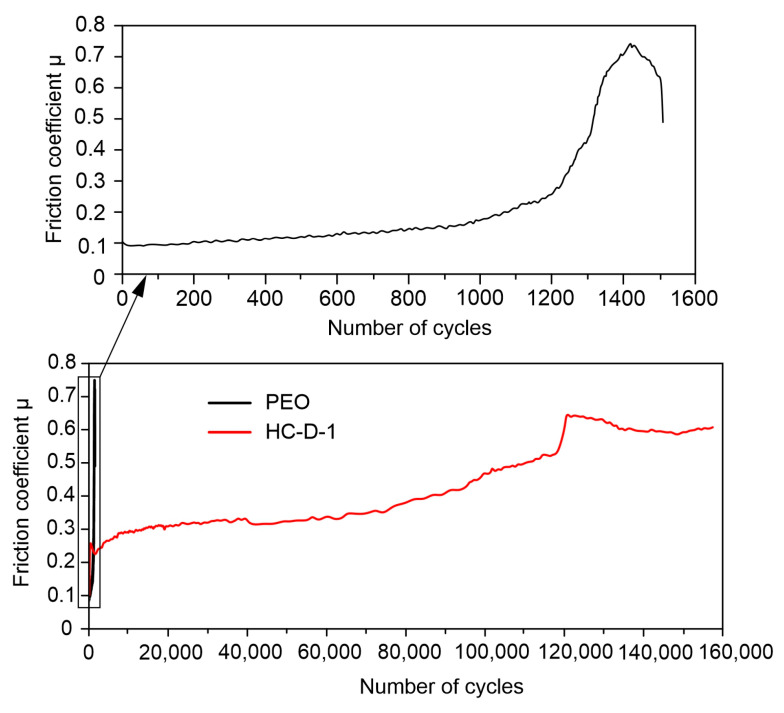
Dependence of the friction coefficient on the number of cycles for samples with different surface treatment.

Analysis of the results of tribological characteristics of the obtained surface layers allowed us to reveal a significant increase in wear resistance after hybrid coating formation (Figure 13, Table 4). Due to the treatment of a PEO-coated sample with polycaprolactone, the coating wear decreased by 25 times.

### 2.5. Self-Healing Mechanism

**Figure 14:**
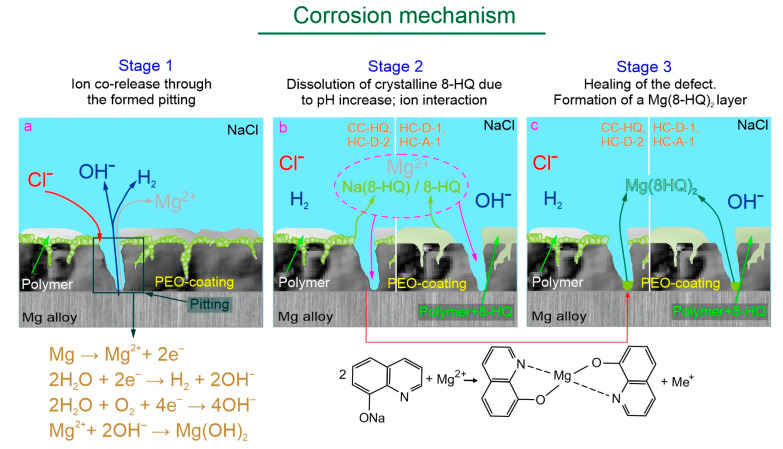
The schema of a self-healing mechanism of the samples with inhibitor-containing coatings. The mechanism includes three stages (**a**–**c**) of the protective film formation.

The schematic interpretation of a self-healing mechanism is presented in Figure 14. The immersion in an aggressive chloride-containing solution contributes to a pitting formation and initiation of Mg substrate corrosion (Stage 1 of the corrosion mechanism) accompanied by the release of Mg^2+^ ions, hydrogen (H_2_) and hydroxide anions (OH^−^). Further exposure of samples with the composite coating (CC-HQ) and hybrid coating (HC-D-2) to NaCl solution is characterized by the dissolution of 8-hydroxyquionoline sodium salt Na(8-HQ) (deposited during the immersion of PEO-coated samples in an inhibitory solution containing NaOH) due to an increase in pH value. For hybrid coatings (HC-D-1, HC-A-1 samples), a release of 8-hydroxyquinoline is observed as a result of local pH increase (Stage 2 of the corrosion mechanism). Therefore, as a result of the ion interaction, the healing of the formed pitting is observed with the formation of the protective Mg(8-HQ)_2_ layer (Stage 3 of the corrosion mechanism). The obtained results are in agreement with the data of [92], where the coating degradation, formation of magnesium 8-hydroxyquinalinate, and re-deposition of Mg_3_(PO_4_)_2_ was shown during the immersion of the PEO-coated Mg sample in NaCl solution-containing 8-HQ.

## 3. Materials and Methods

### 3.1. Substrate Characterization and Pretreatment

Rectangular plates made of MA8 magnesium alloy (wt.%: Mn—1.3–2.2, Ce—0.15–0.35, Mg—balance) with a size of 20 × 30 × 1.5 mm were used as a substrate in this presented study. Before coating, the samples were mechanically ground by means of TwinPrep 5x grinding/polishing machine (Allied High Tech Products, Inc., Compton, CA, USA) using a SiC abrasive paper with a gradual decrease in the abrasive grain size to P1000 grit. The final stages of the pretreatment were rinsing the samples with isopropyl alcohol and drying them in a desiccator at 40 °C.

### 3.2. PEO Coating Formation

The base coatings were formed using a program-controlled plasma electrolytic oxidation unit. Oxidation was carried out in a 3-component electrolyte of the following composition: calcium glycerol phosphate (C_3_H_7_CaO_6_P)—25 g/L, sodium fluoride (NaF)—5 g/L, sodium metasilicate (Na_2_SiO_3_)—7 g/L. The coating was formed in the bipolar polarization mode: the anodic component was maintained potentiostatically at U = 400 V, and the cathodic component was changed galvanodynamically in the current density range from 1.3 to 0.71 A/cm^2^ with the sweep rate of 0.005 A/(cm^2^∙s). The total oxidation time was 120 s, the frequency of the polarization signal was equal to 300 Hz, and the duty cycle was 50%.

### 3.3. Inhibitor and Polymer Treatment

The inhibitory solution was prepared by dissolving 3 g/L 8-HQ in an aqueous alkaline solution (pH = 12.0–12.5). The PEO-coated samples were impregnated with the inhibitory solution by dip-coating method for 2 h under constant stirring.

The 3 wt.% and 6 wt.% polycaprolactone solutions in acetone and dichloromethane were used to determine the optimal concentration for impregnating the porous part of the PEO layer. Solutions for creating hybrid coatings were prepared by dissolving 15 g/L of 8-hydroxyquinoline and polycaprolactone at a concentration of 6 wt.% in acetone and dichloromethane. The increase in the inhibitor concentration is due to its high solubility in organic solvents. Samples with the following types of coatings were prepared for experiments:

PEO—sample with the base coating obtained by plasma electrolytic oxidation;

CC-8HQ—sample with the composite coating obtained by the treatment of a PEO-coated sample in an alkaline solution of 8-hydroxyquinoline;

CC-D—sample with the composite coating obtained by the treatment of a PEO-coated sample in 6 wt.% solution of polycaprolactone in dichloromethane;

CC-A—sample with the composite coating obtained by the treatment of a PEO-coated sample in 6 wt.% solution of polycaprolactone in acetone;

HC-D-2—sample with the hybrid coating obtained in 2 steps by impregnation of a PEO-coated sample in an alkaline solution of 8-hydroxyquinoline, followed by treatment with polycaprolactone dissolved in dichloromethane;

HC-D-1—sample with the hybrid coating obtained in 1 step by treatment of a PEO-coated sample in a solution of polycaprolactone (6 wt.%) and 8-hydroxyquinoline (15 g/L) in dichloromethane;

HC-A-1—sample with the hybrid coating obtained in 1 step by treatment of a PEO-coated sample in a solution of polycaprolactone (6 wt.%) and 8-hydroxyquinoline (15 g/L) in acetone.

To ensure the best filling of the pores of the PEO layer with protective agents, the coating impregnation was carried out using an Epovac vacuum apparatus (Struers, Copenhagen, Denmark). The pressure was decreased to 0.1 bar. After that, the samples were smoothly withdrawn from the solution and dried in a desiccator for 42 h until the organic components of the solvent were completely evaporated. The final stage of the coating process was heat treatment in a muffle furnace at t = 65 °C for 15 min. The polymeric material was applied twice.

### 3.4. SEM/EDX Analysis

The morphology of the obtained coatings and elements distribution over the surface area and thickness of the sample was studied by scanning electron microscopy (SEM) and energy dispersive x-ray analysis (EDX) using a Merlin Gemini 2 device (Carl Zeiss Group, Jena, Germany) with a Silicon Drift Detector X-MaxN 80 (Oxford Instruments NanoAnalysis, Concord, MA, USA). To obtain the cross-sections, the studied samples were embedded with epoxy resin. After that, the obtained tablet was processed by means of a Tegramin-25 grinding and polishing machine (Struers A/S, Copenhagen, Denmark) using grinding paper and polishing cloths with gradual reduction in the abrasive grain size to 3 µm, according to method presented in [93].

### 3.5. Electrochemical Studies (EIS, PDP)

Electrochemical measurements were carried out using the potentiodynamic polarization (PDP), electrochemical impedance spectroscopy (EIS), and open circuit potential (OCP) techniques using the VersaSTAT MC electrochemical system (Princeton Applied Research, Oak Ridge, TN, USA). The tests were performed at room temperature in a three-electrode cell. The 3.5 wt.% NaCl aqueous solution was used as an electrolyte. The area of the exposed sample was equal to 1 cm^2^. The platinized niobium mesh was used as a counter electrode. Monitoring of the electrode potential was carried out vs. silver/silver chloride (Ag/AgCl) reference electrode (the potential versus normal hydrogen electrode is equal to 0.197 V). Before measurements, to stabilize the electrode potential, the samples were kept in the electrolyte for 10 min. The impedance spectrum was recorded in the frequency range from 1 MHz to 0.1 Hz with a logarithmic sweep of 10 points per decade after 10 min, after 1 h exposure of the sample to the electrolyte, and every 2 h for 22 h. Potentiodynamic measurements were carried out at a sweep rate of 1 mV/s. The sample was polarized in the anodic direction in the potential range from *E*_c_ − 0.25 V to *E*_c_ + 0.5 V. To calculate the values of the corrosion potential *E*_c_ and the corrosion current density *I*_c_, the Levenberg-Marquardt approach was used. This method is appropriate for calculating the corrosion parameters of metals with a surface oxide layer, in particular, magnesium and its alloys [41,94]. PDP tests were carried out to assess the ability of coatings to maintain protective properties and self-healing action. PDP measurements were performed after the recording of the impedance spectra (after 10 min and 22 h of sample exposure). Such a technique was previously used in [54]. Calculation of the polarization resistance was carried out using a linear polarization resistance test [40] in a separate experiment. The electrochemical measurements were performed in triplicates for reliability and reproducibility.

### 3.6. Inhibitor Efficiency and Corrosion Rate Evaluation

The effectiveness of the inhibitor (*η_i_*) was evaluated on the basis of calculated parameters obtained from the results of electrochemical tests in accordance with Equation (1).

*η_i_* = (*I_c_*_0_ *− I_c_*)/*I_c_*_0_) × 100%, (1)

where *I_c_*_0_ is the corrosion current density for coatings without a corrosion inhibitor and *I_c_* is the corrosion current density for inhibitor-containing coatings.

The values of the instant corrosion rate (*CR* in mm year^−1^) were calculated using corrosion current density data (in mA cm^−2^) obtained from potentiodynamic polarization measurements according to Equation (2) [41,95].

*CR* = 22.85 × *I_c_*. (2)

### 3.7. Immersion Test

A 7-day immersion test of the coated samples in 3.5 wt.% NaCl solution was performed. For this test, specimens with the size of 15 mm × 20 mm × 1.5 mm were exposed to the 1000 mL of solution at room temperature. At the end of the test, the samples were ultrasonically cleaned in deionized water to remove the corrosion products.

### 3.8. Tribological Studies

The wear resistance of the formed coatings was estimated using a CSM Tribometer (CSM Instruments, Peseux, Switzerland). Tribological tests were performed according to the “ball-on-plate” scheme with a corundum (α-Al_2_O_3_) ball with a 10 mm diameter used as a counterbody. Experiments were carried out under dry friction conditions at room temperature.

The *Wear* (in mm^3^/(N × m)) was calculated in accordance with Equation (3).

*Wear* = ∆*V*/*NF*, (3)

where ∆*V* is the volume of the specimen, which was deleted during the tribological studies, *F* is the applied load, which was equal to 10 N, and *N* is the distance. The volume loss of the specimen was measured as ∆*V* = *S* × *l*, where *S* is the cross-section area and *l* is the track length. The linear rotation speed and track diameter were 50 mm/s and 10 mm, respectively.

## 4. Conclusions

As a result of this study of surface modification of a bioresorbable MA8 alloy (Mg-Mn-Ce system) to reduce the intensity of its corrosion degradation, the following results were obtained.

The bioactive hydroxyapatite-containing coating was formed on the surface of the MA8 alloy by the PEO method. It was established that the morphology of the resulting PEO layer, characterized by the presence of pores and microdefects, is suitable for further loading with a corrosion inhibitor and polymer.

The method of porous PEO coating impregnation with an inhibitor—8-hydroxyquinoline (8-HQ) to increase the corrosion resistance of the processed magnesium alloy was chosen. PEO coating treatment with 8-hydroxyquinoline leads to a reduction of the corrosion current density, *I_c_*, by more than three times, in comparison with a base PEO layer. According to PDP results, the self-healing effect of the inhibitor-containing coating was established after 22 h exposure of the samples to a 3.5% NaCl solution. This is confirmed by a fourfold decrease in the value of *I_c_* compared to the data obtained for the base PEO coating.

To enhance the anticorrosive properties of coatings and retain the inhibitor in the pores of the PEO layer (reducing the spontaneous release of the inhibitor not associated with the corrosion process), the formed layers were modified with a biodegradable polymeric material—polycaprolactone (PCL). Methods for hybrid coatings formation using a system of PCL solutions in dichloromethane or acetone are presented. The optimal concentration of PCL in solutions (6 wt.%) was determined, and the modes of hybrid coatings formation using a combined treatment of PEO layers with PCL and 8-HQ were developed. The method of corrosion inhibitor incorporation (including the number of steps impregnation and the type of solvent) significantly matters to the self-healing mechanism. It was detected that among all the studied coated samples, specimens with hybrid coatings obtained by treatment in a dichloromethane solution containing 6 wt.% of PCL and 15 g/l of 8-HQ are characterized by the highest level of corrosion protection. The inhibitor efficiency for these hybrid coatings was about 80.1–83.9%.

Treatment of a PEO-coated sample with polycaprolactone contributes to a significant increase in the wear resistance of the studied samples.

The detailed mechanism of the self-healing effect of the formed hybrid Ca-P-coatings, which contain a biodegradable polymeric material and a corrosion inhibitor harmless to humans, was established. These protective layers can promote the controlled bioresorption and increase the bioactivity of magnesium-based implant material to its subsequent use in medical practice.

## Figures and Tables

**Table 1:** Electrochemical parameters * obtained during the analysis of polarization curves and impedance spectra after the exposure of the samples to 3.5 wt.% NaCl solution.

Coating Type	*β*_a_, mV/Decade	−*β*_c_, mV/Decade	*I*_c_, A·cm^−2^	*CR*, mm Year^−1^	*E*_c_, V (Ag/AgCl)	*R*_P_, Ω·cm^2^	|*Z*|_*f*=0.1__Hz_, Ω·cm^2^
PEO after 10 min exposure	47.06	239.89	5.61 × 10^−6^	0.128	−1.53	3.05 × 10^3^	2913
PEO after 22 h exposure	75.83	225.22	3.89 × 10^−6^	0.088	−1.41	6.33 × 10^3^	2002
CC-HQ after 10 min exposure	43.41	125.03	1.46 × 10^−6^	0.033	−1.51	9.61 × 10^3^	5838
CC-HQ after 22 h exposure	81.33	76.78	9.56 × 10^−7^	0.022	−1.38	1.80 × 10^4^	3642
CC-D after 10 min exposure	118.76	188.07	1.52 × 10^−6^	0.035	−1.49	2.08 × 10^4^	10,578
CC-D after 22 h exposure	162.73	207.76	1.02 × 10^−6^	0.023	−1.32	3.89 × 10^4^	3566
HC-D-2 after 10 min exposure	127.03	147.03	4.99 × 10^−7^	0.011	−1.57	5.93 × 10^4^	32,338
HC-D-2 after 22 h exposure	201.40	139.23	3.52 × 10^−7^	0.008	−1.37	1.02 × 10^5^	2750
HC-D-1 after 10 min exposure	138.58	155.99	3.02 × 10^−7^	0.007	−1.48	1.06 × 10^5^	105,350
HC-D-1 after 22 h exposure	235.30	97.99	1.64 × 10^−7^	0.004	−1.35	1.83 × 10^5^	15,591
CC-A after 10 min exposure	100.44	168.97	1.74 × 10^−6^	0.039	−1.49	1.58 × 10^4^	11,318
CC-A after 22 h exposure	121.60	224.10	1.77 × 10^−6^	0.040	−1.37	1.94 × 10^4^	3708
HC-A-1 after 10 min exposure	130.11	230.26	5.20 × 10^−7^	0.012	−1.48	6.95 × 10^4^	54,074
HC-A-1 after 22 h exposure	282.98	109.08	6.37 × 10^−7^	0.014	−1.40	5.37 × 10^4^	10,469

* Errors for the measured and calculated parameters did not exceed 10%.

**Table 2:** Calculated parameters * of the equivalent electrical circuit elements for coated samples after the exposure of the samples to 3.5 wt.% NaCl solution.

Exposure Time, h	*CPE*_1_		*R*_1_*,* Ω·cm^2^	*CPE*_2_		*R*_2_*,* Ω·cm^2^
	*Q*_1_*,* S·cm^−2^·s^n^	*n*_1_		*Q*_2_, S·cm^−2^·s^n^	*n*_2_	
*PEO*						
0.17 (10 min)	9.29 × 10^−6^	0.86	134.4	1.17 × 10^−6^	0.86	2527
21.7	1.96 × 10^−5^	0.78	17.8	4.22 × 10^−5^	0.93	1751
*CC-HQ*						
0.17 (10 min)	7.92 × 10^−6^	0.47	423.4	2.90 × 10^−6^	0.87	5123
21.7	6.40 × 10^−6^	0.90	199.7	1.36 × 10^−5^	0.87	2584
*CC-D*						
0.17 (10 min)	1.66 × 10^−9^	0.88	808.5	7.03 × 10^−6^	0.67	10,360
21.7	1.24 × 10^−7^	0.51	165.8	1.26 × 10^−5^	0.80	3817
*HC-D-2*						
0.17 (10 min)	1.14 × 10^−9^	0.89	5360.0	3.39 × 10^−6^	0.65	29,068
21.7	2.62 × 10^−9^	0.93	309.0	1.43 × 10^−5^	0.77	2701
*HC-D-1*						
0.17(10 min)	5.25 × 10^−9^	0.79	3548	1.61 × 10^−6^	0.68	106,450
21.7	2.78 × 10^−9^	0.85	835.5	5.53 × 10^−6^	0.78	12,995
*CC-A*						
0.17 (10 min)	1.63 × 10^−9^	0.88	938.0	7.04 × 10^−6^	0.73	11,114
21.7	1.65 × 10^−8^	0.58	113.0	1.51 × 10^−5^	0.81	3712
*HC-A-1*						
0.17 (10 min)	4.07 × 10^−9^	0.80	1610	1.34 × 10^−6^	0.73	51,048
21.7	4.23 × 10^−8^	0.69	581	5.99 × 10^−6^	0.80	10,167

* Errors for the calculated parameters were <5%. The chi-square value was about χ^2^ = 1 × 10^−4^.

**Table 3:** Inhibitor efficiency * estimated after 10 min and 22 h of exposure to 3.5 wt.% NaCl solution.

Coating Type	CC-HQ	HC-D-2	HC-D-1	HC-A-1
*η_i_.* % (after 10 min exposure)	73.9	67.2	80.1	70.1
*η_i_*. % (after 22 h exposure)	75.4	65.4	83.9	64.0

* Errors for the inhibitor efficiency did not exceed 10%.

**Table 4:** The results of tribological tests.

Sample Type	Number of Cycles	Distance, m	Wear, mm^3^∙(N∙m)^−1^
PEO	1385	48	(1.34 ± 0.30) × 10^−2^
HC-D-1	157,481	5654.7	(5.28 ± 1.00) × 10^−4^

## Data Availability

Not applicable.

## Supplementary Materials

The following supporting information can be downloaded at: https://www.mdpi.com/article/10.3390/molecules28062538/s1, Table S1: Evolution of the calculated parameters of the equivalent electrical circuits elements for a PEO-coated and CC-HQ samples during 22 h of exposure to 3.5 wt.% NaCl; Table S2: Evolution of the calculated parameters of equivalent electrical circuits for CC-D, HC-D-2 and HC-D-1 samples during 22 h of exposure to 3.5 wt.% NaCl; Table S3: Evolution of the calculated parameters of equivalent electrical circuits for a CC-A and HC-A-1 samples during 22 h of exposure to 3.5 wt.% NaCl.
