# Supplementary material for: A Superior Corrosion Protection of Mg Alloy via Smart Nontoxic Hybrid Inhibitor-Containing Coatings
Source: Molecules. 2023 Mar 10;28(6):2538. doi: 10.3390/molecules28062538 (PMC10056050; doi:10.3390/molecules28062538)
Supplement: Supplementary file 1 [file molecules-28-02538-s001.zip › molecules-2252930-supplementary.pdf]

Supplementary Materials

A Superior Corrosion Protection of Mg Alloy via Smart Nontoxic Hybrid Inhibitor-Containing Coatings

Andrey S. Gnedenkov *, Valeriia S. Filonina, Sergey L. Sinebryukhov and Sergey V. Gnedenkov

Institute of Chemistry, Far Eastern Branch of the Russian Academy of Sciences, 159 Pr. 100-letiya Vladivostoka, Vladivostok 690022, Russia

* Correspondence: asg17@mail.com; Tel.: +8-(423)-2215-284; Fax: +8-(423)-2312-590

SUPPLEMENTARY MATERIALS

Table S1. Evolution of the calculated parameters* of the equivalent electrical circuits elements for a PEO-coated and CC-HQ samples during 22 h of exposure to 3.5 wt.% NaCl.

**Exposure time, h**	***CPE*₁**		**R_1, Ω·cm²**	***CPE*₂**		**R_2, Ω·cm²**
	**Q_1, S·cm⁻²·sⁿ**	**n_1**		**Q_2, S·cm⁻²·sⁿ**	**n_2**	
			PEO			
0.17 (10 min)	9.29×10^{-6}	0.86	134.4	1.17×10^{-6}	0.86	2527
1	3.27×10^{-5}	0.79	36.5	6.02×10^{-5}	0.94	1841
3.3	3.57×10^{-5}	0.81	50.5	4.74×10^{-5}	0.95	1886
5.6	3.21×10^{-5}	0.83	48.4	4.46×10^{-5}	0.94	1872
7.9	2.16×10^{-5}	0.82	28.0	4.86×10^{-5}	0.93	1992
10.2	1.82×10^{-5}	0.83	24.7	4.83×10^{-5}	0.93	2000
12.5	2.12×10^{-5}	0.84	29.0	4.38×10^{-5}	0.93	1806
14.8	1.83×10^{-5}	0.82	20.6	4.66×10^{-5}	0.93	1713
17.1	1.98×10^{-5}	0.79	19.6	4.44×10^{-5}	0.93	1752
19.4	2.19×10^{-5}	0.78	19.5	4.29×10^{-5}	0.93	1646
21.7	1.96×10^{-5}	0.78	17.8	4.22×10^{-5}	0.93	1751
			CC-HQ			
0.17 (10 min)	7.92×10^{-6}	0.47	423.4	2.90×10^{-6}	0.87	5123
1	6.66×10^{-6}	0.82	350.0	1.38×10^{-5}	0.87	4071
3.3	7.46×10^{-6}	0.84	294.5	1.21×10^{-5}	0.87	3622
5.6	6.38×10^{-6}	0.87	267.9	1.51×10^{-5}	0.82	3386
7.9	5.15×10^{-6}	0.91	221.7	2.01×10^{-5}	0.78	3351
10.2	6.70×10^{-6}	0.88	200.8	1.23×10^{-5}	0.86	3110
12.5	6.01×10^{-6}	0.89	192.6	1.39×10^{-5}	0.85	3021
14.8	6.01×10^{-6}	0.89	162.3	1.39×10^{-5}	0.85	2971
17.1	7.03×10^{-6}	0.91	169.6	1.29×10^{-5}	0.87	2823
19.4	7.66×10^{-6}	0.88	156.7	1.20×10^{-5}	0.88	2627
21.7	6.40×10^{-6}	0.90	199.7	1.36×10^{-5}	0.87	2584

* Errors for the calculated parameters were < 5 %. The chi-square value was about $\chi^2 = 1 \times 10^{-4}$.

Table S2. Evolution of the calculated parameters* of equivalent electrical circuits for CC-D, HC-D-2 and HC-D-1 samples during 22 h of exposure to 3.5 wt.% NaCl.

**Exposure time, h**	***CPE*₁**		**R_1. Ω·cm²**	***CPE*₂**		**R_2. Ω·cm²**
	**Q_1. S·cm⁻²·sⁿ**	**n_1**		**Q_2. S·cm⁻²·sⁿ**	**n_2**	

CC-D						
0.17 (10 min)	1.66×10^{-9}	0.88	808.5	7.03×10^{-6}	0.67	10,360
1	9.99×10^{-7}	0.51	540.4	8.86×10^{-6}	0.70	10,471
3.3	2.89×10^{-7}	0.61	298.5	9.78×10^{-6}	0.70	5854
5.6	3.92×10^{-7}	0.57	258.3	1.09×10^{-5}	0.74	4406
7.9	4.56×10^{-7}	0.59	205.4	1.03×10^{-5}	0.76	4538
10.2	6.31×10^{-7}	0.55	190.0	9.60×10^{-5}	0.79	3995
12.5	3.32×10^{-7}	0.61	168.9	1.06×10^{-5}	0.79	3835
14.8	1.37×10^{-7}	0.52	176.5	9.75×10^{-5}	0.81	3781
17.1	9.76×10^{-7}	0.54	173.2	1.04×10^{-5}	0.81	3472
19.4	1.07×10^{-7}	0.53	174.3	1.09×10^{-5}	0.81	3352
21.7	1.24×10^{-7}	0.51	165.8	1.26×10^{-5}	0.80	3817
HC-D-2						
0.17 (10 min)	1.14×10^{-9}	0.89	5360.0	3.39×10^{-6}	0.65	29,068
1	1.01×10^{-9}	0.95	737.4	1.12×10^{-5}	0.66	14,112
3.3	9.47×10^{-10}	0.96	655.3	1.47×10^{-5}	0.66	9512
5.6	1.13×10^{-9}	0.95	595.5	1.38×10^{-5}	0.68	7301
7.9	1.27×10^{-9}	0.96	415.6	1.34×10^{-5}	0.71	4390
10.2	1.47×10^{-9}	0.96	360.5	1.46×10^{-5}	0.71	3555
12.5	1.94×10^{-9}	0.94	317.7	1.44×10^{-5}	0.73	3098
14.8	2.30×10^{-9}	0.93	317.6	1.32×10^{-5}	0.75	3354
17.1	1.99×10^{-9}	0.94	307.2	1.42×10^{-5}	0.74	2767
19.4	1.66×10^{-9}	0.96	314.4	1.34×10^{-5}	0.76	2869
21.7	2.62×10^{-9}	0.93	309.0	1.43×10^{-5}	0.77	2701
HC-D-1						
0.17 (10 min)	5.25×10^{-9}	0.79	3548	1.61×10^{-6}	0.68	106,450
1	1.30×10^{-9}	0.89	2824	3.60×10^{-6}	0.67	37,535
3.3	1.48×10^{-9}	0.88	2516	3.93×10^{-6}	0.69	30,947
5.6	1.23×10^{-9}	0.89	1997	5.08×10^{-6}	0.72	15,995
7.9	2.07×10^{-9}	0.86	1326	4.94×10^{-6}	0.74	13,563
10.2	1.96×10^{-9}	0.87	1031	5.28×10^{-6}	0.74	13,946
12.5	2.43×10^{-9}	0.86	982.4	5.06×10^{-6}	0.77	13,180
14.8	2.64×10^{-9}	0.85	952.8	5.44×10^{-6}	0.76	14,068
17.1	2.72×10^{-9}	0.85	905.2	6.29×10^{-6}	0.75	13,494
19.4	2.57×10^{-9}	0.85	889.6	5.46×10^{-6}	0.78	13,739
21.7	2.78×10^{-9}	0.85	835.5	5.53×10^{-6}	0.78	12,995

* Errors for the calculated parameters were < 5 %. The chi-square value was about $\chi^2 = 1 \times 10^{-4}$.

Table S3. Evolution of the calculated parameters* of equivalent electrical circuits for a CC-A and HC-A-1 samples during 22 h of exposure to 3.5 wt.% NaCl.

**Exposure time, h**	***CPE₁***		**R_1, Ω·cm²**	***CPE₂***		**R_2, Ω·cm²**
	**Q_1, S·cm^{-2}·s^n**	**n_1**		**Q_2, S·cm^{-2}·s^n**	**n_2**	
CC-A						
0.17 (10 min)	1.63×10^{-9}	0.88	938.0	7.04×10^{-6}	0.73	11,114
1	2.93×10^{-8}	0.74	368.6	1.05×10^{-5}	0.70	11,102

3.3	3.05×10^{-8}	0.74	312.1	1.05×10^{-5}	0.72	8926
5.6	5.23×10^{-8}	0.68	335.2	1.00×10^{-5}	0.76	8557
7.9	4.41×10^{-8}	0.72	256.8	9.84×10^{-6}	0.77	7496
10.2	3.60×10^{-8}	0.73	237.6	1.05×10^{-5}	0.77	6192
12.5	5.32×10^{-8}	0.71	236.9	9.49×10^{-6}	0.79	7250
14.8	2.94×10^{-8}	0.80	192.3	1.41×10^{-5}	0.80	5321
17.1	2.09×10^{-8}	0.85	107.4	1.42×10^{-5}	0.80	4278
19.4	2.74×10^{-8}	0.74	94.6	1.56×10^{-5}	0.80	3783
21.7	1.65×10^{-8}	0.58	113.0	1.51×10^{-5}	0.81	3712
HC-A-1						
0.17 (10 min)	4.07×10^{-9}	0.80	1610	1.34×10^{-6}	0.73	51,048
1	9.41×10^{-9}	0.77	1601	4.68×10^{-6}	0.69	40,227
3.3	8.97×10^{-9}	0.78	1354	5.51×10^{-6}	0.69	30,362
5.6	1.02×10^{-8}	0.77	1100	7.21×10^{-6}	0.68	19,613
7.9	1.05×10^{-8}	0.78	865	6.62×10^{-6}	0.72	13,778
10.2	1.08×10^{-8}	0.78	729	6.60×10^{-6}	0.73	11,151
12.5	1.51×10^{-8}	0.76	670	5.95×10^{-6}	0.75	11,181
14.8	2.25×10^{-8}	0.73	645	6.08×10^{-6}	0.76	10,485
17.1	2.16×10^{-8}	0.74	602	6.04×10^{-6}	0.77	10,962
19.4	3.85×10^{-8}	0.70	597	5.92×10^{-6}	0.79	10,426
21.7	4.23×10^{-8}	0.69	581	5.99×10^{-6}	0.80	10,167

* Errors for the calculated parameters were < 5 %. The chi-square value was about $\chi^2 = 1 \times 10^{-4}$.
